# Altered splicing leads to reduced activation of CPEB3 in high-grade gliomas

**DOI:** 10.18632/oncotarget.9735

**Published:** 2016-05-31

**Authors:** Magdalena Skubal, Gerrit H. Gielen, Anke Waha, Marco Gessi, Lech Kaczmarczyk, Gerald Seifert, Dorothee Freihoff, Johannes Freihoff, Torsten Pietsch, Matthias Simon, Martin Theis, Christian Steinhäuser, Andreas Waha

**Affiliations:** ^1^ Institute of Cellular Neurosciences, Medical Faculty, University of Bonn, 53105 Bonn, Germany; ^2^ Institute of Neuropathology, Medical Faculty, University of Bonn, 53105 Bonn, Germany; ^3^ German Center for Neurodegenerative Diseases (DZNE), 53127 Bonn, Germany; ^4^ Institute of Neurosurgery, Medical Faculty, University of Bonn, 53105 Bonn, Germany

**Keywords:** CPEB, glioma, DNA methylation, splicing, expression

## Abstract

Cytoplasmic polyadenylation element binding proteins (CPEBs) are auxiliary translational factors that associate with consensus sequences present in 3′UTRs of mRNAs, thereby activating or repressing their translation. Knowing that CPEBs are players in cell cycle regulation and cellular senescence prompted us to investigate their contribution to the molecular pathology of gliomas–most frequent of intracranial tumors found in humans. To this end, we performed methylation analyses in the promoter regions of *CPEB1-4* and identified *the CPEB1* gene to be hypermethylated in tumor samples. Decreased expression of CPEB1 protein in gliomas correlated with the rising grade of tumor malignancy. Abundant expression of CPEBs2-4 was observed in several glioma specimens. Interestingly, expression of CPEB3 positively correlated with tumor progression and malignancy but negatively correlated with protein phosphorylation in the alternatively spliced region. Our data suggest that loss of CPEB3 activity in high-grade gliomas is caused by expression of alternatively spliced variants lacking the B-region that overlaps with the kinase recognition site. We conclude that deregulation of CPEB proteins may be a frequent phenomenon in gliomas and occurs on the level of transcription involving epigenetic mechanism as well as on the level of mRNA splicing, which generates isoforms with compromised biological properties.

## INTRODUCTION

Control of mRNA polyadenylation is an important step in regulation of gene expression. Cytoplasmic polyadenylation element binding (CPEB) proteins activate or inhibit translation of mRNA molecules by binding to the consensus CPE sequences present in the 3′untranslated regions of transcripts [1, 2]. In the cytoplasm, CPEBs assemble into a ribonucleoprotein (RNP) complex consisting of Gld2 Poly(A) Polymerase, Poly(A)-specific ribonuclease (PARN), Poly(A) binding protein (ePAB), eIF4E binding protein (Maskin), RNA binding complex (CPSF) and a scaffold protein (symplekin). In basal conditions, when the activity of PARN deadenylase exceeds Gld2 polymerase, CPEBs promote translational repression by holding the bound mRNAs in a dormant state. Following phosphorylation and subsequent PARN dissociation, CPEB proteins initiate elongation of mRNA poly(A) tails and thereby stimulate translation [3–5].

The family of CPEB proteins was first identified in the gametogenesis of Xenopus [6, 7] where CPEB1 was shown to be involved in the control of meiosis [8, 9]. Recent findings indicate that CPEB1 contributes to various biological processes like synaptic plasticity [10], mitotic cell cycle control [11, 12] and cellular senescence of murine [13] or human fibroblasts [14]. Furthermore, inhibition of CPEB1 activity results in the Warburg effect characterized by metabolic changes in cells, like elevated glycolysis, reduced cellular respiration and generation of reactive oxygen species at constant ATP content [14]. Therefore the active state of CPEB is crucial for proper cell metabolism. The mechanisms regulating stimulation of CPEBs and subsequent translation of mRNA molecules involve phosphorylation of CPEB1 by Aurora A or Ca^2+^/calmodulin-dependent protein kinase II (CaMKII) [15–17].

In contrast to CPEB1 much less is known about CPEB 2-4 proteins and their alternative splice variants. Spliced regions of CPEB1 were described to have low similarity with CPEB2-4 [18]. Sequences of CPEB2-4 are highly similar in RNA recognition motifs (RRMs) but variable in their N-termini. Nevertheless, the 8-aa motif (B-region) localized in the N-terminus and overlapping with kinase binding sites is conserved [19]. Its deletion leads to the removal of functional motifs for phosphorylation, protein-protein interactions and post-translational modifications [19]. The isoforms of CPEB2-4 differ in the presence or absence of the B-region adjacent to corresponding tyrosine and serine/threonine binding sites regulating protein activity. Protein kinase A (PKA) and Ca^2+^/calmodulin-dependent protein kinase II (CaMKII) were shown to phosphorylate CPEB2-4 [18–22]. It was hypothesized that alternative splicing of CPEBs modify the function of proteins [19, 21], but so far the biological importance of this phenomenon has been rather neglected.

We have previously shown that CPEB1 and CPEBs2-4 are broadly expressed in mouse brain [18, 19, 21]. Taking into account the function of CPEB proteins, it is conceivable that dysregulation of respective CPEB regulated transcripts may cause modifications in cellular properties e.g. cellular metabolism, signal transduction, motility, proliferation or viability, i.e. parameters known to contribute to malignant transformation [23]. Therefore, in this study we investigated whether CPEB family members are involved in the molecular pathology of gliomas. To this end, we determined the expression and activity of CPEB proteins as well as their activating kinases. Furthermore, we investigated splice variants of CPEB family members known to activate CPEBs in a cohort of gliomas retrieved from the German Brain Tumor Reference Center. A lack of CPEB3 activation was observed in high grade gliomas suggestive of defective translational control in these tumors. Given their important role in the regulation of protein expression, our findings put into medical attention the elucidation of target transcripts and the cellular pathways altered by CPEBs.

## RESULTS

### Methylation in the 5‘-CpG-island of CPEB1-4 genes

**Figure 1:**
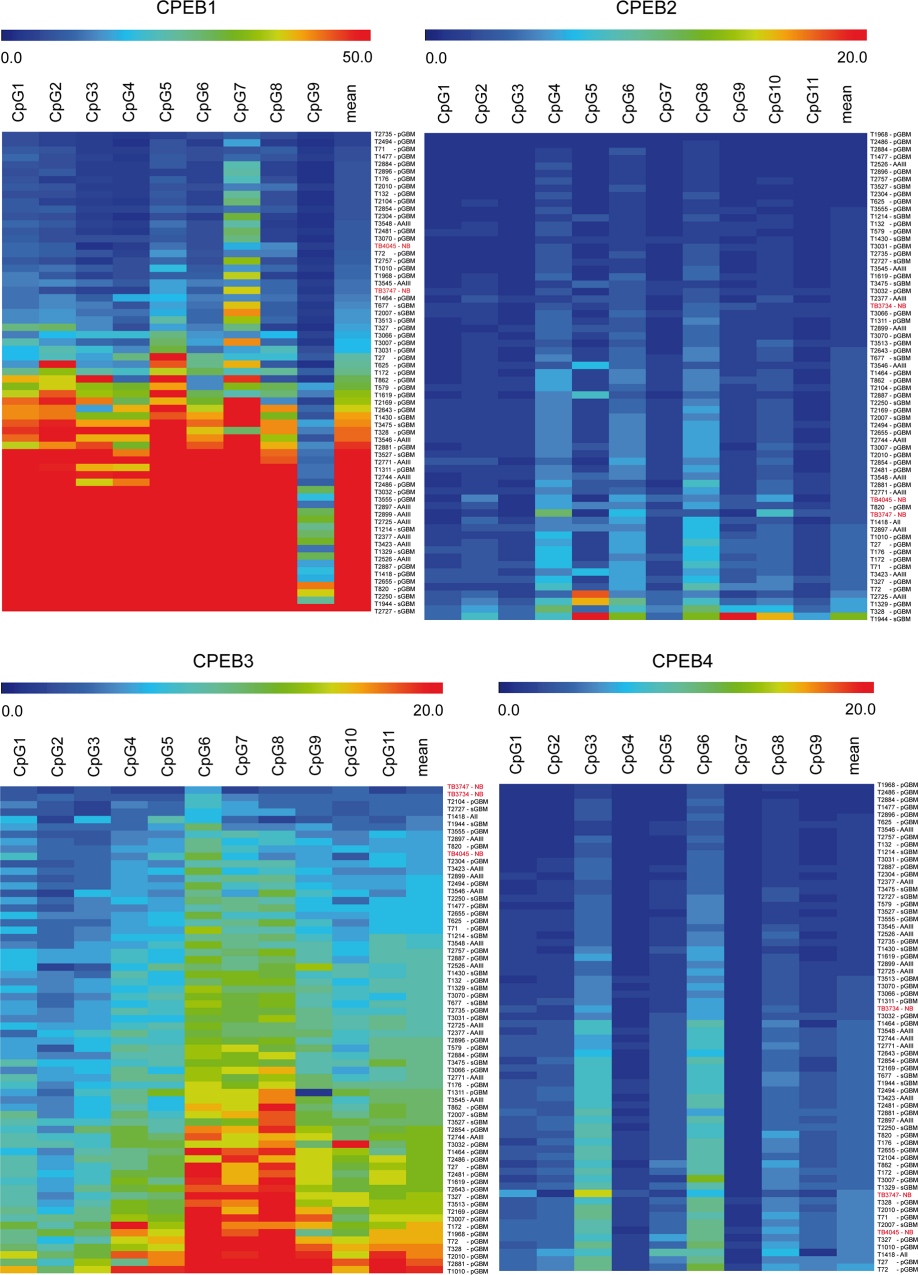
Methylation profile of *CPEB1-4* genes in glioma and reference tissue measured by pyrosequencing Scale above heat maps displays the specific methylation areas in % (range 0–50% for *CPEB1* and 0–20% for *CPEB2-4*). Columns correspond to the investigated CpG dinucleotides, whereas rows to the individual glioma (WHO grade II–IV, *n* = 63) and control normal brain (NB, labeled in red, *n* = 3) tissue samples. Blue color on heat map indicates lack of methylation, while red corresponds to increased methylation of CpG sites in investigated tumors.

We have identified *CPEB1* as a target for epigenetic inactivation by differential methylation hybridization (DMH). By pyrosequencing we investigated the methylation levels of *CPEB1* as well as the other members of the CPEB family in 63 human glioma, 3 normal brain samples (Figure 1) and 5 glioblastoma cell lines (data not shown). Normal brain tissue of age-matched patients showed only trace methylation of up to 16% in the investigated CpG-islands (Supplementary Figure S1). As a cut-off level for methylation we chose three fold the standard deviation of mean methylation of normal brain samples. *De novo* methylation of *CPEB1* was observed in the majority of AAIII (9/11). Within the group of GBM a strong *CPEB1* hypermethylation was especially abundant in tumors that developed following malignant progression of lower-grade precursor lesions (sGBM: 10/10). Secondary GBM tumors containing the *IDH1* mutation (*n* = 7) revealed a mean methylation of 69.37 ± 6.78%. Our cohort of pGBM (*n* = 41) samples contained 4 cases with *IDH1* mutation, which also revealed a significant increase of *CPEB1* methylation (mean 73.53 ± 4.26%). Secondary GBM without *IDH1/2* mutation (*n* = 3) and primary GBM tissues with wild type *IDH1/2* (*n* = 37) showed a mean methylation of 21.81 ± 8.93% and 19.84 ± 2.74% in the investigated region of *CPEB1*, respectively. This indicates that *CPEB1* methylation is tightly linked to the *IDH1* mutation status. In addition, all investigated glioblastoma cell lines showed hypermethylation of the *CPEB1* gene. The observed methylation pattern shows that *CPEB1* belongs to the genes affected by the glioma associated CpG island methylator phenotype (G-CIMP) in *IDH1/2* mutant tumors. Correlation of *IDH1/2* mutation with *CPEB1* methylation was highly significant (Fisher's two-sided exact test, *p* < 0.001). Compared to CPEB1, methylation levels of CPEB3 were low (*n* = 61, mean methylation of 10.19 ± 0.43%) in the entire cohort of samples, and only a few cases showed moderately elevated methylation. There was no correlation of *CPEB3* methylation, expression and *IDH1/2* mutation. For *CPEB2* and *CPEB4* no methylation was detected in any of the investigated tumor specimens (Figure 1).

### Characterization of CPEB1-4 expression in glioma tissues

**Figure 2:**
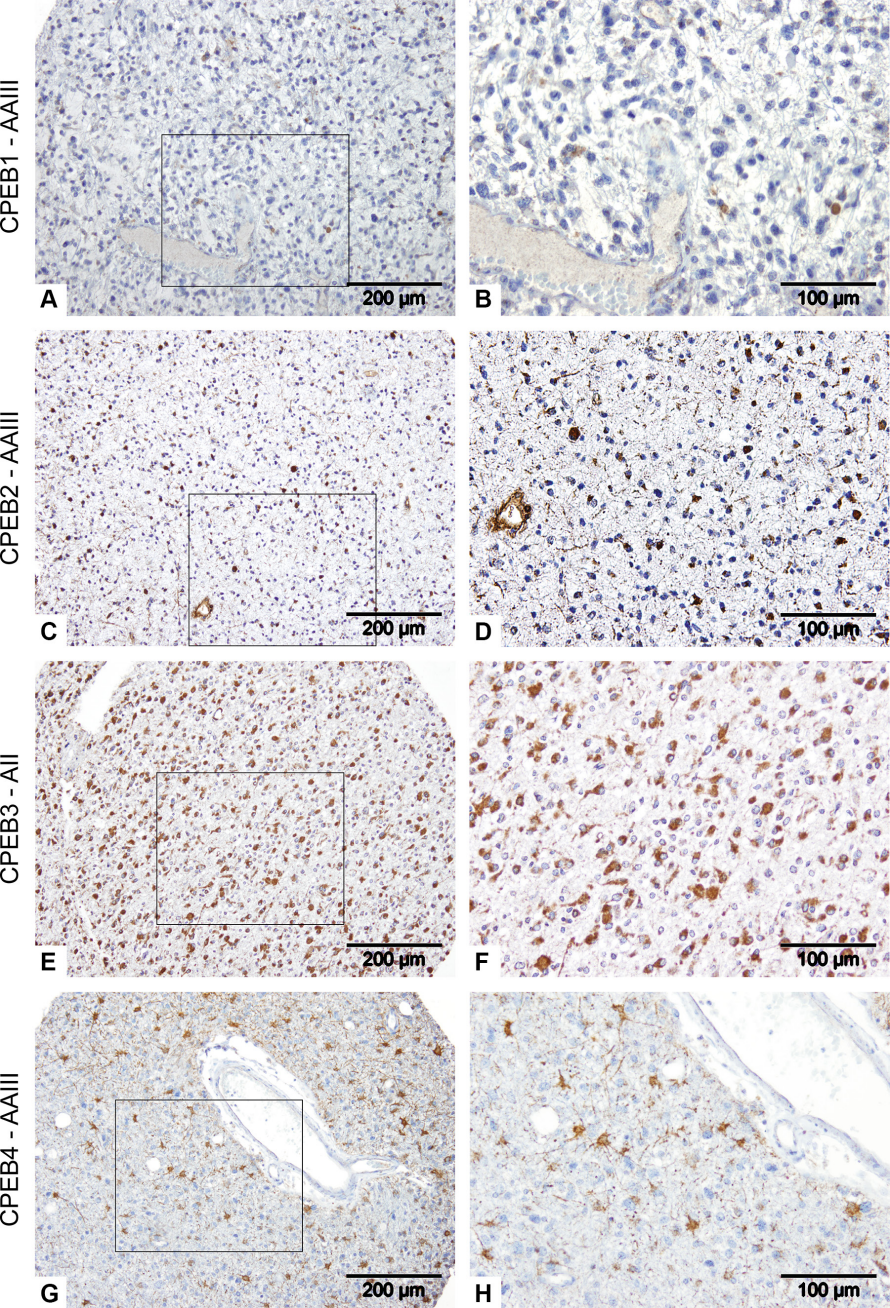
Analysis of CPEB protein expression in gliomas Immunohistochemical staining of AAIII (**A–D**, **G, H**) and AII (E, F) specimens. A, B: CPEB1; C, D: CPEB2; E, F: CPEB3 and G, H: CPEB4. Brown areas represent CPEBs immunoreactivity, while blue hematoxylin staining corresponds to cell nuclei.

**Figure 3:**
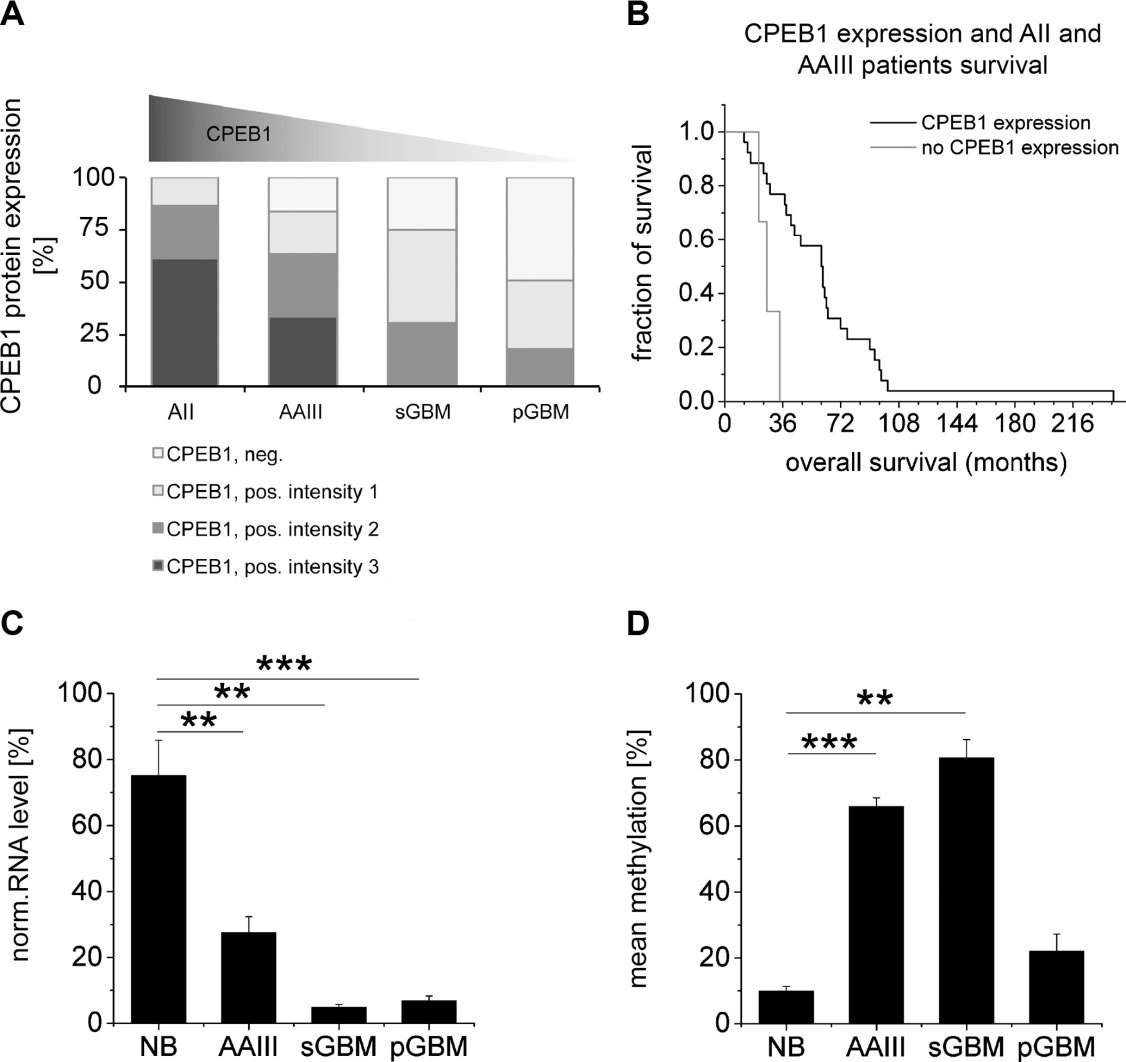
Comparison of *CPEB1* methylation and expression with glioma patient survival (**A**) Downregulation of CPEB1 protein expression was observed with rising glioma malignancy. Strong expression of CPEB1 was restricted to low-grade astrocytoma samples, while most of the GBMs showed low CPEB1 immunoreactivity. Light grey graph sections correspond to the lack of CPEB expression, while grey and dark grey sections to increasing CPEB staining divided into intensity groups (1: weak, 2: intermediate, 3: strong). (**B**) Correlation between expression of CPEB1 and patient survival. Kaplan- Meier survival analysis showed a correlation between CPEB1 expression in low-grade gliomas and longer survival of patients (*n* = 29, *p* < 0.01). (**C**) Compared to non-tumor control samples (NB, *n* = 6), a significant reduction of CPEB1 transcript was observed in AAIII (*n* = 6, *p* < 0.01), sGBM (*n* = 3, *p* < 0.01) and pGBM (*n* = 16, *p* < 0.001). Error bars indicate standard error of the mean. (**D**) Methylation of *CPEB1* gene was significantly increased in AAIII (*n* = 6, *p* < 0.001) and sGBM (*n* = 3, *p* < 0.01), but not in pGBM, when compared to normal brain samples. Error bars indicate standard error of the mean.

Tissue microarrays containing a total of 69 glioma specimen in duplicates were used for a histological characterization of CPEB1-4 proteins expression (Figure 2). Our studies revealed that all CPEB proteins were present in glioma tissues and were characterized by a distinctive and differential staining pattern and intensity. Strong CPEB1 expression was detected in few (2/61) tumor specimens and was located in the infiltration areas of tumor cells into healthy brain tissue (Supplementary Table S2). The vast majority of cells in the tumor center, in the areas of necrosis and vascular proliferation showed no CPEB1 expression. We observed decrease of CPEB1 protein expression with rising grade of glioma malignancy (Figure 3A). Most of the astrocytoma specimens showed staining for CPEB1 (26/29: 8/8 AII and 18/21 AAIII), while 23/32 glioblastoma (6/7 sGBM and 17/25 pGBM) samples contained CPEB1 positive cells (Supplementary Table S2, Figure 3A). Expression of CPEB2 was present in the majority of studied tumors (8/9 AII; 18/20 AAIII; 7/8 sGBM; 18/25 pGBM) (Supplementary Table S2). CPEB2 could be detected in reactive astrocytes in normal brain tissue and in endothelial cells of blood vessels within the tumors (Figure 2). CPEB3 expression appeared to be the most abundant amongst CPEBs in gliomas and present in cytoplasm and processes of astrocytic tumor cells (gemistocytes) (Figure 2, Supplementary Table S2). Strong CPEB3 staining of tumor cells was observed in 8/10 AII, 19/20 AAIII, 7/7 sGBM and 23/24 pGBM (Supplementary Table S2). Enhanced immunoreactivity against CPEB3 was associated with increasing grade of malignancy. In contrast to CPEB3 expression, phosphorylation of CPEB3 protein was observed mainly in low-grade gliomas (7/8 AII, 17/20 AAIII) and distinctly lower in glioblastomas (10/26 pGBM) (Figure 6A, Supplementary Table S2). CPEB4, analogous to CPEB1 was strongly expressed (10/62) in only few tumors. CPEB4 positive tumor cells often showed a strong enhancement of protein expression in cell processes (Figure 2, Supplementary Table S2).

Furthermore, expression of CPEB1 transcript was studied in 6 normal brain and 25 glioma samples by sqRT-PCR (Figure 3C). All investigated tumor tissues (AAIII *n* = 6, sGBM *n* = 3, pGBM *n* = 16) revealed a significant reduction of CPEB1 transcript when compared to reference samples. Expression of CPEB1 mRNA was decreased to 36.63 ± 6.52% in AAIII (*p* < 0.01), to 6.36 ± 1.23% and 9.05 ± 1.90% in sGBM (*p* < 0.01) and pGBM (*p* < 0.001), respectively (Figure 3C). Methylation of the *CPEB1* gene was analyzed in the identical set of samples. A significant increase in methylation was detected in AAIII (*p* < 0.001) and sGBM (*p* < 0.01), but not in pGBM (Figure 3D). Methylation and RNA levels of *CPEB1* were compared by Pearson's correlation. No correlation was found between *CPEB1* mRNA content and *CPEB1* methylation for any of the tumor subtypes tested (data not shown).

### Alteration of cancer-associated signaling pathways by CPEB1

**Figure 4:**
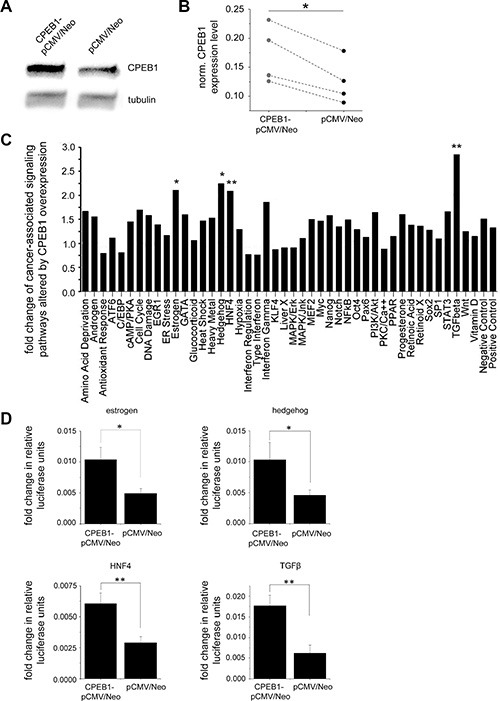
Alternation of cancer-associated signaling pathways by CPEB1 (**A, B**) Transient overexpression of CPEB1-pCMV6/Neo or control pCMV6/Neo plasmid in A172 cultured glioblastoma cells monitored by immunoblot (*n* = 4, *p* < 0.05). (**C**) Quantitative analysis of cancer associated signaling pathways investigated by pathway reporter array (*n* = 3). Diagram depicts the change of reporter activity indicative of specific cancer-related pathways altered by CPEB1 overexpression. Fold change was obtained by dividing the pathways activity upon CPEB1-pCMV6/Neo expression by activity upon control pCMV6/Neo plasmid. (**D**) Statistical quantification of estrogen (*n* = 3, *p* < 0.05), hedgehog (*n* = 3, *p* < 0.05), HNF4 (*n* = 3, *p* < 0.01) and TGFβ (*n* = 3, *p* < 0.01) pathways. Error bars indicate standard error of the mean.

Alteration of individual cancer relevant signaling pathways upon CPEB1 overexpression was investigated by a cell based reporter assay. CPEB1 protein was transiently expressed in glioblastoma A172 cultured cells and its transfection efficiency was monitored by Western blot analysis (Figure 4A, 4B). The analysis revealed a significant upregulation of estrogen (*n* = 3, *p* < 0.05), hedgehog (*n* = 3, *p* < 0.05), HNF4 (*n* = 3, *p* < 0.01) and TGFβ (*n* = 3, *p* < 0.01) pathways (Figure 4C, 4D).

### Identification of alternative splice isoforms of CPEB1-4

**Figure 5:**
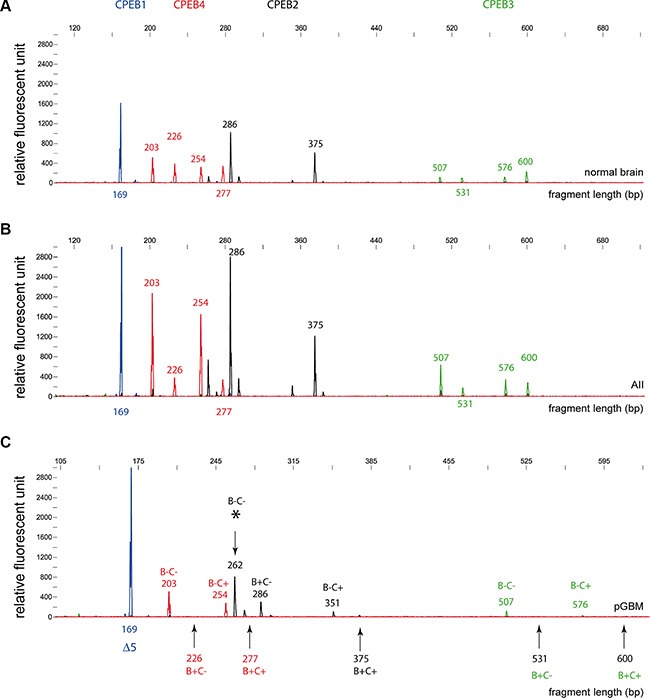
Fragment analysis of CPEB alternative splice variants in gliomas and normal brain specimens RT-PCR analysis revealed the abundance of respective splice variants of the four CPEB genes. Values on the x-axis correspond to the length of the RT-PCR product generated for the fragment analysis in base pairs. Vertical y-axis defines signal intensity (relative fluorescent units), which is proportional to the amount of generated PCR product. The size of the RT-PCR product generated for the fragment length analysis did not describe the actual length of the respective splice product. Differences between splice variants are due to presence or absence of respective B- or C-regions. (**A**) CPEB isoforms in normal brain tissue are: CPEB1Δ5 (169 bp); CPEB2c (286 bp; lack of C and E-region), CPEB2a (375 bp; full isoform), CPEB3d (507 bp; lack of B and C-region), CPEB3c (531 bp; lack of C-region), CPEB3b (576b; lack of B-region), CPEB3a (600bp; full isoform); CPEB4d (203bp; lack of B and C-region), CPEB4c (226 bp; lack of C-region), CPEB4b (254 bp; lack of B-region), CPEB4a (277 bp; full isoform of CPEB4). (**B**) The splice variants detected in normal brain were also present in AII/AAIII gliomas. (**C**) In GBM samples, loss of several CPEB2, CPEB3 and CPEB4 (arrows) splice variants was observed. Interestingly, GBM samples displayed a unique CPEB2 variant (asterisk) that only showed a trace signal in normal brain. Additional information on the respective splice variants is given in Supplementary Figure S2 and Supplementary Tables S4 and S5.

The relative abundance of CPEB1-4 splice variants was analyzed by RT-PCR fragment analysis (Figure 5). We detected a significant change in splicing between tumor (*n* = 58) and normal brain tissue (*n* = 4). The majority of the examined AII cases contained the same splice variants as normal brain tissue (Figure 5A, 5B). In GBM samples we observed a loss of several of the CPEB 2, 3 and 4 variants that were present in normal brain, while the CPEB1Δ5 (169 bp) variant was present in all cases (Figure 5C, Supplementary Table S5). In nearly all pGBM (*n* = 35) and in all normal tissues analyzed (*n* = 4), CPEB1Δ5 (169 bp) was the only CPEB1 isoform that was detected. Two pGBM samples (cases 3070 and 3555) showed an additional CPEB1 (182 bp) splice variant, which corresponds to the full-length isoform (Supplementary Table S5, Supplementary Figure S2). Interestingly, insertion or deletion of the 5-aminoacides (Δ5) in RRM1 of CPEB1 correlates with specificity of RNA binding [19].

In the investigated cohort of samples, the CPEB2- 4 isoforms containing the B-region and being a target for phosphorylation were often depleted, while isoforms lacking the B-region were expressed. In the vast majority of GBM specimens CPEB3a (36/37 pGBM, 8/8 sGBM) and CPEB4a (30/37 pGBM, 6/8 sGBM) disappeared, while those isoforms were still present in AAIII tumors and normal brain tissues. Almost all of the GBM samples (35/37 pGBM and 6/8 sGBM) were devoid of the CPEB4c splice variant which also contains the B-region (Supplementary Figure S2, Supplementary Tables S4, S5). Additionally, we found that some of the detected CPEB2 isoforms contain the E-region, which was previously described in mouse CPEB2 [21].

### Upregulation of CPEB3 protein in gliomas

**Figure 6:**
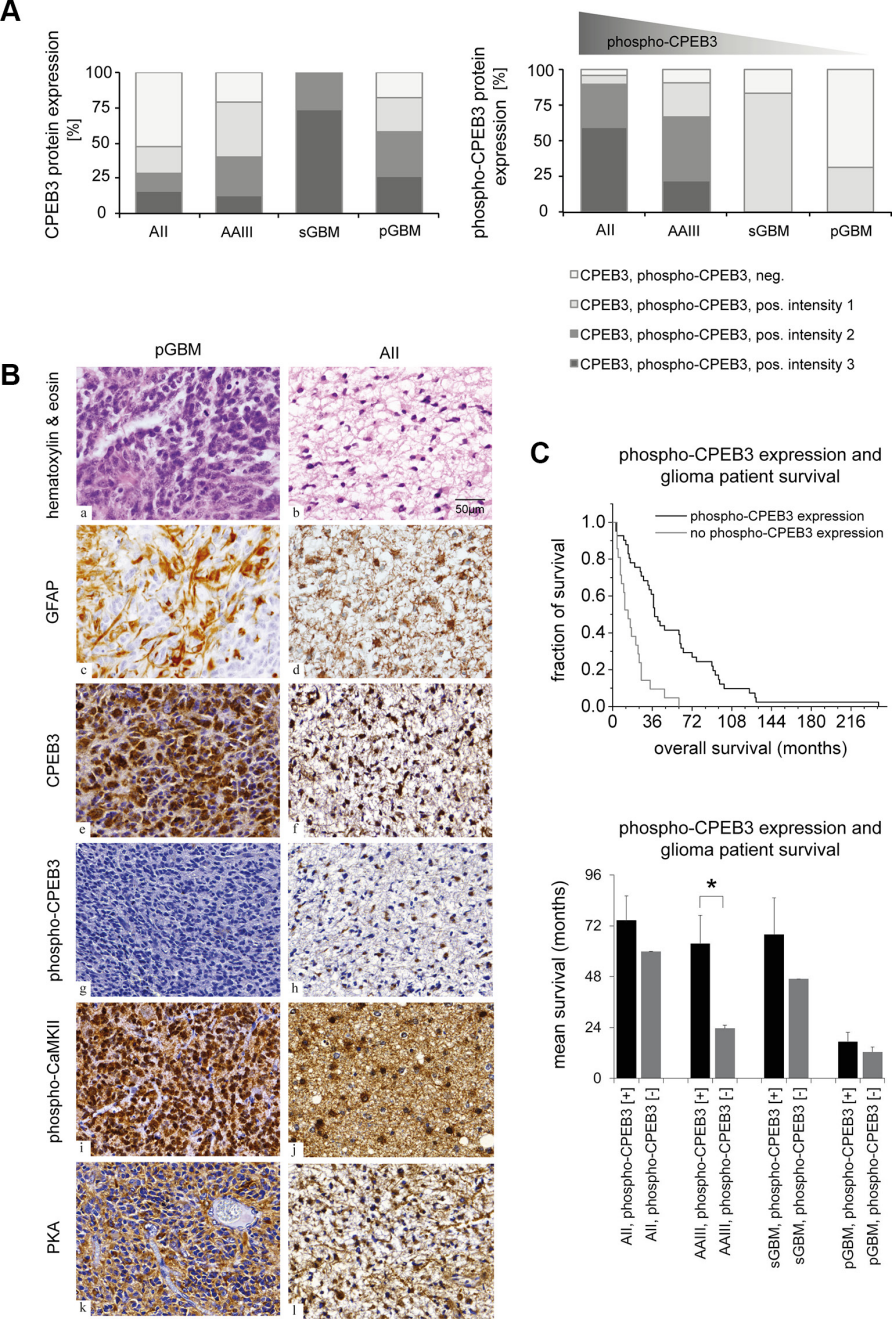
Altered CPEB3 activity in gliomas and its correlation with overall patient survival (**A**) Evaluation of CPEB3 and phospho-CPEB3 protein expression in gliomas. Light grey graph sections correspond to the lack of CPEB expression, while grey and black sections to CPEB expression. Immunoreactivity was divided into intensity groups (1: weak, 2: intermediate, 3: strong). Upregulation of CPEB3 expression and reduction of phospho-CPEB3 activity was observed in parallel to the growing grade of glioma malignancy. (**B**) Expression of CaMKII and PKA kinases induce the activation of CPEB3 protein in gliomas. Representative staining of pGBM: a, c, e, g, i, k and AII: b, d, f, h, j, l tissue samples. Pictures a, b present cell nuclei stained with violet hematoxylin, and cell cytoplasm stained with pink eosin. On the remaining pictures (c-l), dark blue areas represent cell nuclei stained by hematoxylin, while brown areas correspond to respective antibody staining: GFAP: c, d; CPEB3: e, f; phospho-CPEB3: g, h; phospho-CaMKII: i, j; PKA: k, l. (**C**) Correlation between phospho-CPEB3 expression and overall patient survival observed in all investigated glioma samples (*p* < 0.001, *upper panel*). The positive link between phospho-CPEB3 expression and prolonged mean patient survival was particularly evident for AII (*n* = 8), AAIII (*n* = 20, *p* < 0.01) and sGBM (*n* = 8) (Figure 6C- *lower panel*). Error bars indicate standard error of the mean.

Immunohistochemical analysis of CPEBs in gliomas indicated that expression of active phospho-CPEB3 protein shows an inverse correlation with the glioma malignancy grade (Figure 6A, right). In order to understand why CPEB3 protein is strongly expressed but not activated/phosphorylated in high-grade gliomas, we decided to further examine tumors with strong (intensity 3) phospho-CPEB3 expression (*n* = 6, AII and AAIII) and samples negative for phospho-CPEB3 (*n* = 7, pGBM). All these samples revealed strong immunoreactivity againstCPEB3 protein in the cytoplasm and processes of astrocytic tumor cells. We investigated the expression/activity of kinases that were shown before to phosphorylate CPEB3 protein [18, 19, 21, 22]. PKA and CaMKII kinases showed predominant activity in human astrocytomas, which were also positive for phospho-CPEB3 (Figure 6B). Interestingly, both kinases were also abundantly expressed in GBM specimens lacking CPEB3 phosphorylation (Figure 6B) with only moderate reduction of CaMKII kinase in 4/6 samples (data not shown).

### Correlation between molecular findings and clinical data

An overall survival (OS) time was defined as the time between surgery for the primary tumor and death of the glioma patient. Patients alive at the time of their last follow-up were surveyed. A total number of 61–62 samples (Supplementary Table S2) were included in the Kaplan-Meier survival analysis comparing CPEB expression with patient survival. The Kaplan-Meier curve suggests a positive correlation between CPEB1 protein expression in 29 astrocytoma specimens and longer survival of patients (Figure 3B, Log-rank (Mantel-Cox) test *p* < 0.01). Within the group of patients with pGBM, 17/25 showed CPEB1 protein expression, but there was no correlation with survival (data not shown). The group of sGBM contained only 7 cases, so no statistic evaluation was performed. No correlation between CPEB2, CPEB3 and CPEB4 proteins expression and patient survival was observed in the examined AII, AAIII and GBM samples (data not shown). The activation of CPEB3, as determined by immunostaining, significantly correlated with survival in all investigated tumor samples (Figure 6C-*upper panel*, *p* < 0.001). The differences in survival of patients with and without phospho-CPEB3 expression were particularly obvious for AII (*n* = 8, 7/8 samples contained phospho-CPEB3), AAIII (*n* = 20, 17/20 samples contained phospho-CPEB3, *p* < 0.01) and sGBM (*n* = 8, 7/8 samples contained phospho-CPEB3) (Figure 6C- *lower panel*). This finding indicates that phospho-CPEB3 can be considered an independent biological marker for a better prognosis in astrocytomas with a similar pathogenesis. In contrast, there was no such prognostic correlation for pGBM (*n* = 26, 10/26 samples contained phospho-CPEB3) (Figure 6C- *lower panel*).

## DISCUSSION

Differential expression of cancer-related genes has been widely investigated in gliomas. Results of these experiments allow today for more precise and timely molecular-based diagnosis of tumors. Microarray expression profiling based on transcript analysis allowed molecular subtyping of gliomas with identification of predictive and prognostic markers [24–26]. Recent reports indicate that post-transcriptional modifications of specific mRNA populations regulate the expression changes of genes under physiological conditions, but may also contribute to the molecular pathology related to tumorigenesis [27, 28]. Therefore, translational regulation by polyadenylation is an important mechanism in the development and progression of cancer and may represent a new therapeutic avenue. Here, we investigated the expression of CPEB isoforms in human glioma specimens and glioma cell lines to address their potential contribution to the pathology of brain tumors.

Gliomas are the most frequent form of intracranial neoplasms and are believed to derive from neuroepithelial tissue. The WHO divides them into astrocytomas, oligodendrogliomas, mixed gliomas (oligoastrocytomas) and ependymomas. Within the group of astrocytomas, glioblastoma multiforme (GBM) is the most frequent (50%) malignant tumor. The greatest numbers of GBM (90%) called pGBM develop *de novo*. The remaining GBM cases develop by progression of lower-grade precursor lesions to sGBM. Astrocytomas make up the largest group of gliomas and show a diffuse infiltrative growth into normal brain tissue. Median survival of patients diagnosed with GBM remains less than 12 months [29, 30]. Therefore, the dramatic situation of GBM cases and lack of appropriate effective treatment indicate that it is essential to learn more about the molecular pathology of these tumors in order to develop new diagnostic and therapeutic approaches and improve the clinical course of these patients. The first indication of altered CPEB protein expression in human glioma came from our genome wide DNA methylation analyses. Dysregulation of *CPEB* methylation was previously investigated in tumor tissue [31, 32], but until now hypermethylation of *CPEB* genes in glioma has not been reported. Here, we localized a DNA fragment upstream of the *CPEB1* gene and developed a pyrosequencing assay to quantitatively assess the methylation of this position in a cohort of 63 human glioma specimens obtained from the German Brain Tumor Reference Center. Hypermethylation of *CPEB1* was observed in gliomas containing a mutant *IDH1* gene. Mutant IDH1 proteins produce the oncometabolite, 2-hydroxyglutarate (2-HG) and lead to a decrease of α-ketoglutarate (α-KG), inhibiting the activity of α-KG dependent oxygenases, which are involved in demethylation of genomic DNA and histone tails. This ultimately leads to altered epigenetic marks e.g. the glioma hypermethylator phenotype (G-CIMP) Besides *CPEB1*, we only observed altered methylation of a few CpG sites in the *CPEB3* gene. No correlation of *CPEB3* methylation with *IDH1* mutation was observed.

Subsequently, we determined the abundance and localization of CPEB proteins in glioma tissue by immunohistochemistry. CPEB1 was present in the infiltration areas of tumor cells into normal brain tissue. The vast majority of cells in the tumor center, in the areas of necrosis and vascular proliferation showed no CPEB1 expression, suggesting that expression of CPEB1 is silenced along with progression of cancer (Figures 2, 3B). CPEB2 protein was detected in reactive astrocytes, in normal brain tissue and in endothelial cells of tumors, indicating that stimulation of protein synthesis occurs in the direct proximity of blood vessels in newly formed tumor tissue (Figure 2). CPEB3 was the most abundantly expressed CPEB family member in human gliomas. Its protein content was increasing with WHO grade, reaching highest levels in GBM while activation of the protein, as determined by its phosphorylation, was decreasing with tumor grade (Figure 6A). Accordingly, expression of active phospho-CPEB3 protein was a main feature of low-grade gliomas (Figure 6A). On the other hand, CPEB4, analogous to CPEB1, was highly expressed only in a few tumors, with tumor cells often showing strong immunoreactivity in cell processes (Figure 2).

Since *de novo* methylation is considered as a mechanism of transcriptional silencing we investigated the expression of CPEB1 and compared our findings with the clinical data (Figure 3A, 3B). Interestingly, in the group of low-grade astrocytoma patients, prolonged survival was correlated with the amount of protein expression (Figure 3B). Downregulation of CPEB1 on the transcript as well as protein level was associated with a rising grade of glioma malignancy (Figure 3A, 3C). A significant decrease of CPEB1 transcript was detected in AAIII and sGBM samples which contained *IDH1* mutations and significantly increased *CPEB1* methylation (Figure 3C, 3D). Interestingly, pGBM, containing wild type *IDH1* and being completely devoid of *CPEB1* methylation also revealed reduced expression of CPEB1 transcript (Figure 3C, 3D). Obviously, beside DNA methylation other mechanisms may be relevant for transcriptional repression of CPEB1 in gliomas (Figure 3C, 3D).

The multipathway activity assay carried out to investigate the influence of CPEB1 overexpression on the activity of cancer-associated signaling pathways, revealed an upregulation of estrogen, hedgehog, HNF4 and TGFβ pathways in glioblastoma cells (Figure 4C, 4D). Previous studies indicated that, depending on the type and stage of tumor, TGFβ can promote or suppress tumor progression [33, 34]. It acts as an inhibitor of proliferation in astrocytes, epithelial and immune cells. In order to escape a cytostatic response, most of the tumors accumulate mutations in this pathway. However, there is also data suggesting that TGFβ may act as an oncogenic factor in gliomas by promoting cell proliferation, invasion, angiogenesis and inhibition of immune response [35–38]. A recent report employing CPEB1 mutant lacking relevant phosphorylation sites showed that TGFβ treatment reduces CPEB1 mRNA [39], which independently confirms a link between the TGFβ signaling pathway and CPEB1 expression. However, further studies are needed to fully elucidate the interplay between CPEB1 translational regulation and the activation of the TGFβ signaling cascade.

The obvious difference between expression levels of CPEB3 and active CPEB3 tempted us to investigate CaMKII and PKA kinases, which are known to phosphorylate CPEB proteins [18, 22]. Interestingly, both kinases were abundantly expressed in almost all investigated tumor specimens (Figure 6B). Accordingly, lack of active kinases cannot explain the loss of CPEB3 activation in high-grade gliomas. Therefore, we performed profiling of splicing variants that have been described for CPEB genes and observed a shift towards isoforms lacking the B-region, which is necessary for efficient CPEB3 phosphorylation (Supplementary Table S5). This interesting finding suggests that by alternative splicing, high-grade glioma cells achieve a deregulation of CPEB3-dependent translational control (Figure 5, Supplementary Table S5). Although it is currently unclear how this shift in the splicing process is established, it is interesting to note that a similar link has been observed in CPEB1, where the mutant protein lacking regions of phosphorylation could not be activated and kept bound mRNAs in translational arrest [40]. Additionally, we observed correlation between expression of active CPEB3 protein and clinical data. Our findings indicate that phospho-CPEB3 may be considered as a marker for prolonged survival of glioma patients, especially for patients diagnosed with AII, AAIII and sGBM that share a similar molecular background.

Altered gene expression is a major contributor to the molecular pathogenesis of cancer and determines the pathophysiology and phenotype of the transformed cell. Even though there is firm evidence that this deregulation is attributed to the initiation of transcription, other regulatory processes may also contribute to altered gene expression in cancer cells. Given the fact that CPEB proteins control the translation of various genes, the identification of these transcripts should be a major future task to better understand their contribution to the molecular pathology of cancer. Our finding that CPEB3 in high grade gliomas is expressed as a splice variant that cannot be activated by phosphorylation suggests that it might contribute to altered protein expression in these tumors and be considered an attractive target for new therapeutic strategies.

## MATERIALS AND METHODS

### Tumor specimens and glioblastoma cell lines

Glioma tumor specimens were obtained from 69 patients (26 female, 43 male) treated at the University of Bonn Medical Center, including: 28 patients with primary glioblastoma (WHO grade IV, pGBM), 8 patients with secondary glioblastoma (WHO grade IV, sGBM), 22 patients with anaplastic astrocytoma (WHO grade III, AAIII) and 11 patients with diffuse astrocytoma (WHO grade II, AII). Tumors were graded according to the World Health Organization (WHO) classification of tumors of the central nervous system [41]. Histologic evaluation confirmed that all specimens used for extraction of nucleic acids consisted of a minimum of 80% tumor cells. Each specimen was treated in an anonymous manner as approved by the ethics committee at the University of Bonn. Extraction of DNA and RNA from tumor samples was carried out using ultracentrifugation of homogenized tumor tissue through a CsCl gradient as described before [42].

Glioblastoma cell lines A172, A178, LN229, U373MG, U87MG and T98G were received from the Ludwig Institute for Cancer Research (San Diego). Extraction of RNA from the cultured glioblastoma cells was carried out using RNeasy Mini Kit (Qiagen, Hilden, Germany) following the manufacturer's protocol. Residual genomic DNA was removed by digestion with RNase-free DNase (Roche, Mannheim, Germany). Human normal white matter tissue was used as a reference. The identity of the individual cell lines has been confirmed by STR DNA profiling of 15 loci plus sex-determining marker amelogenin (Genetica DNA Laboratories, Cincinnati, USA).

### Analysis of CPEB 1-4 by bisulfite treatment and pyrosequencing

Genomic DNA of 63 glioma tissues, 5 glioblastoma cell lines and 3 reference white matter tissues was treated with sodium bisulfite using the EpiTect Bisulfite kit (Qiagen, Hilden, Germany). Primers used for amplification of bisulfite treated DNA and primers used for pyrosequencing are given in Supplementary Table S1. Pyrogram outputs were analyzed using the PyroMark Q24 software. Values obtained by pyrosequencing were imported into MeV: Multi Experiment Viewer and visualized as a heat map.

### Reverse transcription and identification of alternative CPEB1-4 splice isoforms

To identify alternative splice variants cDNA synthesis was done using 1 μg of total RNA, random hexamers and SuperScript II Reverse Transcriptase (Invitrogen, Darmstadt, Germany). Detection of distinct splice variants of CPEB1-4 was performed using RT-PCR with primers spanning previously defined splice variants. Primers for RT-PCR, product length, as well as information about the amplified splice variants are summarized in Supplementary Table S4. One of the primers of each pair was labeled with a fluorescent dye allowing sensitive and simultaneous detection and sizing of the PCR products on an automated DNA sequencing machine 3130 (Applied Biosystems/PerkinElmer). PCR products were generated in separate PCR reactions optimized for the individual PCR product, diluted to adjust optimal signal strength and then loaded as pooled products for each individual tumor specimen or cell line. Electropherogramms of the individual samples were analyzed using the GeneMapper v3.7 software (Applied Biosystems/PerkinElmer).

### Generation of antibodies against CPEB1, CPEB2, CPEB4 and phospho-CPEB3

Rabbit polyclonal CPEB1, CPEB2, CPEB4 antibodies and phosphospecific antibody directed to CPEB3 a/c isoforms were generated as described before [21, 22]. For generation of antibodies the following peptides were synthesized: CPEB1: RGIHDQLPDFQDSEETVT; CPEB2: LQLPAWGSDSLQDSWC; phospho-CPEB3: RRGRSSLFPFED and CPEB4: KPPSPWSSYQSPSPTP.

### Tissue microarray analysis and immunohistochemical staining

Tumor tissues were dissected, fixed with 4% phosphate-buffered formalin for 24–48 h at 4°C and submerged in paraffin wax. The arrays were assembled by taking core needle biopsies from preexisting paraffin-embedded tissue blocks and re-embedding these tissues in an arrayed master block. 4 μm thick tissue sections were pretreated in the Lab Vision PT Modul (Thermo Scientific, Fremont, USA) and in PT Modul Buffer (pH 6) (Medac, Hamburg, Germany) for 20 min at 99°C, followed by a cool-down phase of 20 min at room temperature. Immunohistochemical staining was performed with CSA II Biotin-free Tyramide Signal Amplification System (Dako, Carpinteria, USA). To reduce high non-specific background staining, endogenous peroxidases were blocked by incubation in peroxidase blocking buffer for 5 min followed by 60 min protein block at room temperature. Incubation with primary antibodies was performed overnight at 4°C. The following primary antibodies were used: CPEB1 (1:100, custom-made [21]), CPEB2 (1:250, custom-made [21]), CPEB3 (1:100, Abcam, ab10833), CPEB4 (1:250, custom-made [21]). In addition we have incubated the tissue microarrays with a phosphorylation specific antibody detecting the active forms of CPEB3, CaMKII and PKA proteins: phospho-CPEB3 (1:50, custom-made [22]), phospho- CaMKII (1:1000, Cell Signaling, 3361) and PKA (1:500, Abcam, ab59218). Signal detection was performed using the subsequent reagents: polymer (Poly-HRP-Goat anti-mouse/-rabbit IgG), amplification reagent, anti-fluorescein-HRP and 3,3′-diaminobenzidine (DAB) for 15 min each. Nuclei were stained with hematoxylin (Merck, Darmstadt, Germany). Additional immunohistochemical (IHC) analysis with an antibody against GFAP (1:1000, Dako, M0761) was performed on a semi-automated IHC Stainer (Tecan, Crailsheim, Germany).

### Transfection of CPEB1 expression vectors into glioma cell lines

Human glioblastoma A172 cells were grown in DMEM with 25 mM glucose supplemented with 10% fetal calf serum (Gibco, Darmstadt, Germany), 1% penicillin/streptomycin (Gibco, Darmstadt, Germany), 200 mM L-glutamine (Gibco, Darmstadt, Germany) and 100 mM sodium pyruvate (Gibco, Darmstadt, Germany) at 37°C, 5% CO_2_. CPEB1-pCMV6/Neo plasmid and pCMV6/Neo control vector were designed and purchased from OriGene (Rockville, USA). A172 cells were seeded in 6-well plates and transfected with 2 μg of CPEB1-pCMV6/Neo or pCMV6/Neo vector using 3 μl Lipofectamine 3000 transfection reagent (Life Technologies, Carlsbad, USA) for each reaction. After 48 h of incubation, cells were collected for RNA and protein analysis.

### Quantitative real-time PCR

Isolated mRNAs were reversely transcribed with SuperScript II reverse transcriptase (Life Technologies, Carlsbad, USA). One μg of total RNA template and 250 ng of random hexamers for priming, in a 20 μl total reaction volume were used. Real-time PCR was performed using the TaqMan Universal PCR Master Mix (Applied Biosystems, *Warrington*, *UK*). To achieve a semi-quantitative assessment, 2 μl of cDNA was amplified with 900 nM of each CPEB1 and PBGD primers and 100 nM of fluorogenic Taqman probes labeled with 6-FAM fluorophore (5′-end) and TAMRA quencher (3′-end) in a 12.5 μl final reaction volume (Supplementary Table S3). Every PCR was carried out in duplicates. For each reaction, a critical threshold cycle (C_T_) value was determined using SDS 5.0 Software (Applied Biosystems). Normalization was performed using the housekeeping gene PBGD as a reference against the expression of CPEB1 gene transcripts. The transcript level of CPEB1 and the reference gene was calculated using the following equation [43]: X_CPEB1_/X_PBGD_ = 2^CT PBGD – CT CPEB1^, where X are the respective input copy numbers and C_T_ the threshold cycle numbers for CPEB1 and PBGD.

### Western blot analysis

Cell culture lysates were prepared in a modified RIPA lysis buffer supplemented with phosphatase and protease inhibitors (Thermo Scientific, Rockford, USA). First, cells were scraped in ice-cold buffer and incubated on ice for 30 min. Supernatants were collected after 15 min centrifugation at 14.000 g at 4°C. Afterwards, the total protein content was determined by a BCA Protein Assay (Thermo Scientific, Rockford, USA). Lysates were then mixed with denaturing sample buffer and heated for 10 min at 65°C. Next, the protein samples (30 μg) were separated by SDS-PAGE under denaturing conditions and blotted on a PVDF membrane. Membranes were blocked with 5% milk powder in TBS-T buffer, pH 7.4 and incubated overnight at 4°C with primary antibodies. For detecting horseradish peroxidase activity, Supersignal West Dura Substrate (Thermo Scientific, Rockford, USA) was used. Generated chemiluminescence was detected with the Gene Genome digital documentation system (Synoptics, Cambridge, UK). Densitometry with data analysis was performed with GeneTools System (Synoptics) and tested for significant differences.

### Multipathway activity assay

The cell based 45-Pathway Reporter Array (SA Biosciences, Frederick, USA) allows for the quantitative analysis of cancer-associated signaling pathways, by screening their activities. A172 cells overexpressing CPEB1-pCMV6/Neo or pCMV6/Neo vector were reverse transfected with reporter constructs from the reporter array plate. Each reporter assay was composed of an inducible transcription factor responsive *firefly luciferase* reporter and a constitutively expressed *Renilla* construct. As positive and negative controls non-inducible *firefly luciferase* reporter and constitutively expressed *Renilla* constructs, and a mixture of a constitutively expressed GFP construct mixed with a constitutively expressed *firefly luciferase* and *Renilla luciferase* construct were used, respectively. Reverse transfection was performed following the manufacturer's recommendation (SA Biosciences). 48 h post reverse transfection, a luciferase activity was analyzed by Dual-Luciferase Reporter Assay (Promega, Madison, USA). The results were expressed as fold change, calculated by dividing the normalized luciferase activities of each pathway-focused reporter reverse transfected into A172 cells overexpressing CPEB1-pCMV6/Neo plasmid by the normalized luciferase activity of each pathway-focused reporter reverse transfected with the control pCMV6/Neo vector. Reporters showing at least two-fold change in relative luciferase units were considered upregulated or downregulated. Experiments were performed three times in duplicates and analyzed using GloMax 96 Microplate Luminometer (Promega, Madison, USA).

### Data analysis

Histological characterization of tissue microarrays was performed by microscopic observation and digital scan of stained specimens. Tissues were classified as positive or negative for the respective antibody. Afterwards, the positive samples were further divided into three intensity groups: 1-weak, 2-intermediate or 3-strong. Statistical analysis (Student's *t* test or Fisher's exact test) of CPEB expression, *CPEB1/3* methylation as well as putative correlation with clinical and molecular data including overall survival, malignancy grade, *IDH* status was carried out with Prism Software Version 6 the GraphPad Software, Inc (La Jolla, USA) and OrigeneLab Corporation (Northampton, USA).

## SUPPLEMENTARY FIGURES AND TABLES



## Abbreviations

CaMKII: Ca2+/calmodulin-dependent protein kinase II
CPE: Cytoplasmic Polyadenylation Element
CPEB: Cytoplasmic Polyadenylation Element Binding protein
CPSF: Cleavage and Polyadenylation Specificity Factor
DMH: Differential Methylation Hybridization
ePAB: Poly(A) Binding Protein
G-CIMP: glioma-CpG Island Methylator Phenotype
HNF4: Hepatocyte Nuclear Factor 4
IDH1: Isocitrate Dehydrogenase 1
OS: Overall Survival
PARN: Poly(A)-specific ribonuclease
PKA: Protein Kinase A
RNP: Ribonucleoprotein complex
TGFβ: Transforming Growth Factor β
WHO: World Health Organization
