# Supplementary material for: Altered splicing leads to reduced activation of CPEB3 in high-grade gliomas
Source: Oncotarget. 2016 May 31;7(27):41898–912. doi: 10.18632/oncotarget.9735 (PMC5173104; doi:10.18632/oncotarget.9735)
Supplement: Supplementary file 1 [file oncotarget-07-41898-s001.pdf]

Altered splicing leads to reduced activation of CPEB3 in high-grade gliomas

Supplementary Materials

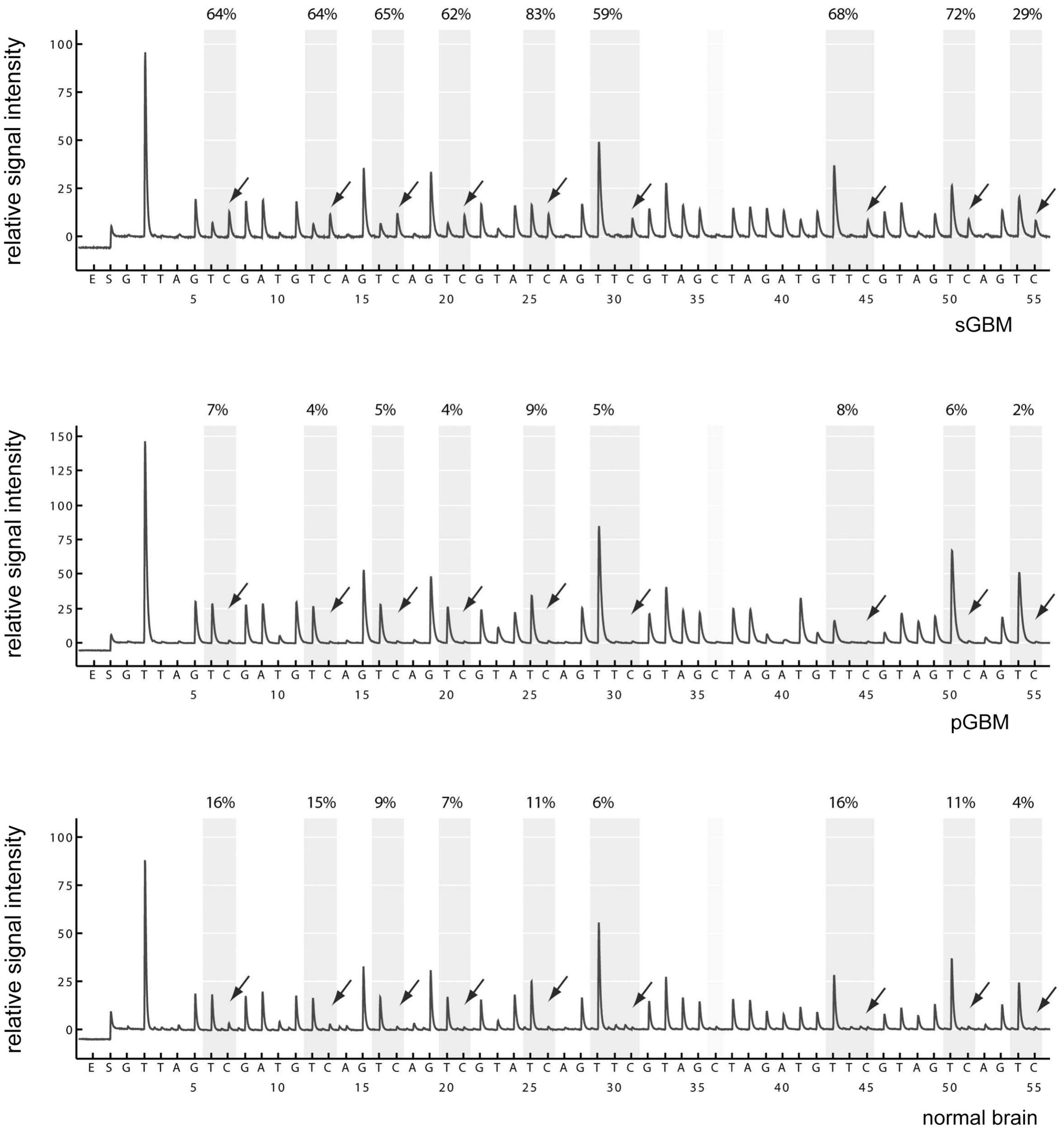

Supplementary Figure S1: Pyrosequencing analysis of CpG islands in the 5′-region of CPEB1 gene in glioma and normal brain tissue. The pyrogram of sGBM (top) showed strong methylation of up to 83% of the CpG positions. The pyrograms of a pGBM (middle) and normal brain tissue (bottom) showed methylation of the investigated region up to 16%.

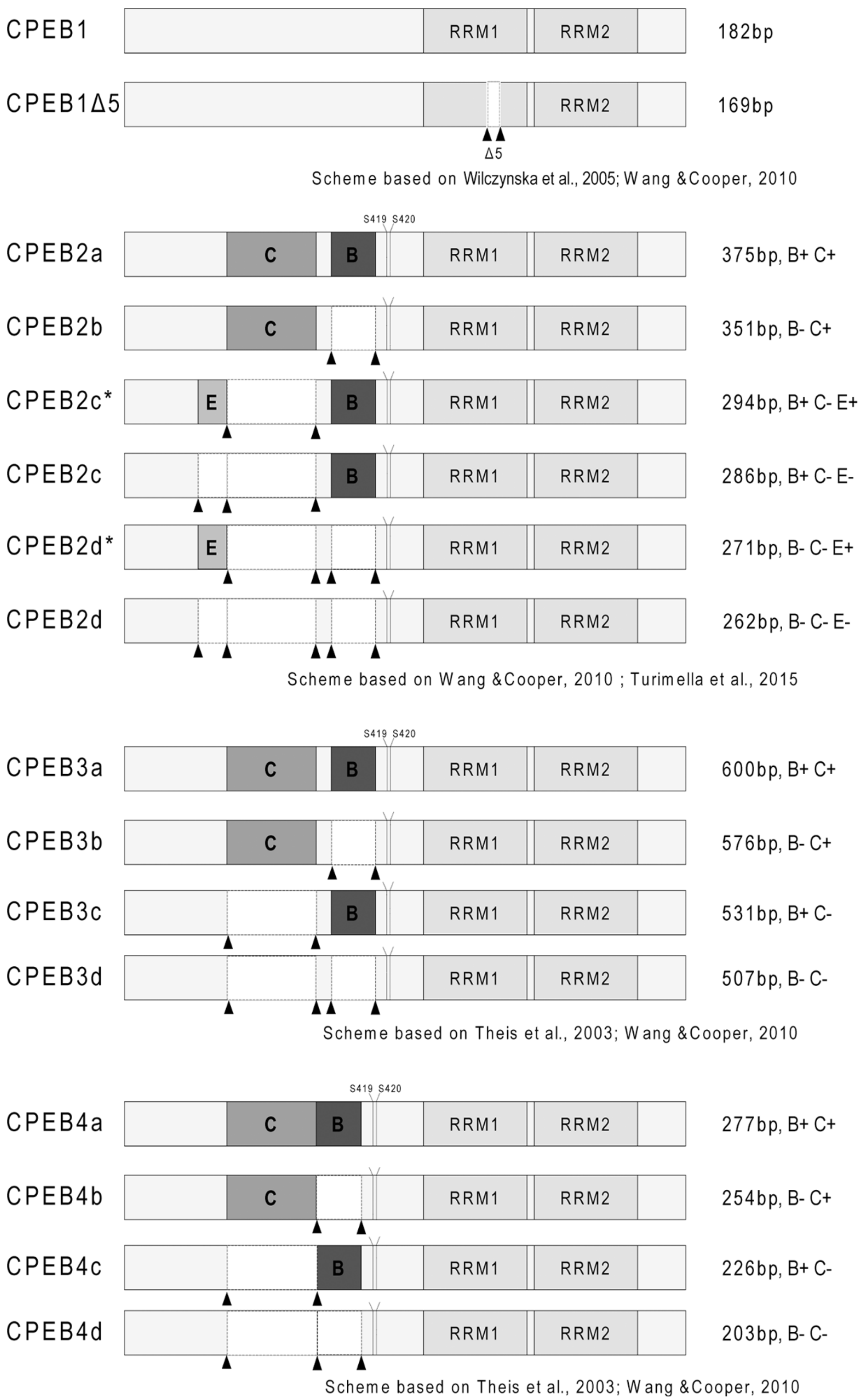

Supplementary Figure S2: Scheme displaying alternative splice variants of CPEB1-4 detected by fragment analysis of human glioma samples. Primers flanking the variable region resulted in 2 (CPEB1), 4 (CPEB3-4) and 6 (CPEB2) amplicons with varying sizes. The Δ5 deletion of CPEB1, full size isoforms of CPEB2-4 and alternations in the presence of B- C- E- regions of CPEB2-4 were labeled accordingly.

Supplementary Table S1: Primers used for amplification of bisulfite treated DNA and primers used for pyrosequencing

Primers for amplification of bisulfite treated DNA:

Gene	*Sequence*
CPEB1	fw 5′ GGGGGTTAGAGATTTAAGTTTGAG 3′
CPEB1	rev 5′-BIOTIN-ACTCCCATCCAAAAAAAACCAATAATATCT 3′
CPEB2	fw 5′ GGGGGTTATTAGTTTAAGTGAGAGTG 3′
CPEB2	rev 5′-BIOTIN-TCCCCTACCCAAATTCACT 3′
CPEB3	fw 5′-BIOTIN-GGGGGTTATTAGTTTAAGTGAGAGTG 3′
CPEB3	rev 5′ ACCACCAACCCATCATAAC 3′
CPEB4	fw 5′ GGGGAAAAGAGAGAGAAAGT 3′
CPEB4	rev 5′-BIOTIN-ACTTCCTCTCCCCCATAA 3′

"fw"and "rev" mark forward and reverse primers.

Primers for pyrosequencing:

Gene	*Sequence*
CPEB1	ps 5′-AAGAGGGTAAGATTTATAAG-3′
CPEB2	ps 5′-TGGGGGAGTGGGAGA-3′
CPEB3	ps 5′-CCAACCCATCATAACC-3′
CPEB4	ps 5′-GGTTTTAGTATTTTTAG-3′

"ps" marks pyrosequencing primers.

Supplementary Table S2: Evaluation of CPEB1-4 expression in glioma tissue

Expression of CPEB1 in:	*no. of n.a. samples*	*no. of investigated samples*	*no. of negative samples*	*no. of positive samples*		
				intensity 1 (weak)	*intensity 2 (intermediate)*	*intensity 3 (strong)*
low-grade astrocytoma (AII)	3	8	0	3	4	1
anaplastic astrocytoma (AAIII)	1	21	3	8	9	1
secondary glioblastoma (sGBMIV)	1	7	1	4	2	0
primary glioblastoma (pGBMIV)	3	25	8	12	5	0
content as sum:	8	**61**	**12**	**27**	**20**	**2**
content as percentage:		**100%**	**19.67%**	**44.26%**	**32.79%**	**3.28%**

Expression of CPEB2 in:	*no. of n.a. samples*	*no. of investigated samples*	*no. of negative samples*	*no. of positive samples*		
				intensity 1 (weak)	*intensity 2 (intermediate)*	*intensity 3 (strong)*
low-grade astrocytoma (AII)	2	9	1	2	2	4
anaplastic astrocytoma (AAIII)	2	20	2	9	4	5
secondary glioblastoma (sGBMIV)	0	8	1	1	4	2
primary glioblastoma (pGBMIV)	3	25	7	10	6	2
content as sum:	7	**62**	**11**	**22**	**16**	**13**
content as percentage:		**100%**	**17.74%**	**35.48%**	**25.81%**	**20.97%**

Expression of CPEB3 in:	*no. of n.a. samples*	*no. of investigated samples*	*no. of negative samples*	*no. of positive samples*		
				intensity 1 (weak)	*intensity 2 (intermediate)*	*intensity 3 (strong)*
low-grade astrocytoma (AII)	1	10	2	3	3	2
anaplastic astrocytoma (AAIII)	2	20	1	8	9	2
secondary glioblastoma (sGBMIV)	1	7	0	0	3	4
primary glioblastoma (pGBMIV)	4	24	1	6	12	5
content as sum:	8	**61**	**4**	**17**	**27**	**13**
content as percentage:		**100%**	**6.56%**	**27.87%**	**44.26%**	**21.31%**

Expression of phospho-CPEB3 in:	*no. of n.a samples*	*no. of investigated samples*	*no. of negative samples*	*no. of positive samples*		
				intensity 1 (weak)	*intensity 2 (intermediate)*	*intensity 3 (strong)*
low-grade astrocytoma (AII)	3	8	1	2	3	2
anaplastic astrocytoma (AAIII)	2	20	3	10	6	1
secondary glioblastoma (sGBMIV)	0	8	1	7	0	0
primary glioblastoma (pGBMIV)	2	26	16	10	0	0
content as sum:	7	**62**	**21**	**29**	**9**	**3**
content as percentage:		**100%**	**33.87%**	**46.77%**	**14.52%**	**4.84%**

Expression of CPEB4 in:	*no. of n.a. samples*	*no. of investigated samples*	*no. of negative samples*	*no. of positive samples*		
				intensity 1 (weak)	*intensity 2 (intermediate)*	*intensity 3 (strong)*
low-grade astrocytoma (AII)	1	10	0	6	3	1
anaplastic astrocytoma (AAIII)	2	20	0	12	5	3
secondary glioblastoma (sGBMIV)	1	7	1	1	4	1
primary glioblastoma (pGBMIV)	3	25	4	11	5	5
content as sum:	7	**62**	**5**	**30**	**17**	**10**
content as percentage:		**100%**	**8.06%**	**48.39%**	**27.42%**	**16.13%**

Investigated tissues were classified as being positive or negative for the respective antibody. Immunoreactivity was subdivided into three intensity groups: 1-weak, 2-intermediate and 3-strong n.a. labels not accessible samples.

Supplementary Table S3: Primers and probes used for semi quantitative real-time PCR

Gene	*Sequence*	*Position*	*Product length*	*GeneBank accession no.*
CPEB1	fw 5′ GCACCCAGGACTCAGATTCC 3′	335(v1), 110 (v2)	73 bp (v1),	NM_030594
CPEB1	rev 5′ CCCAGTGGGTTATGGAGCAT 3′	388 (v1), 163 (v2)	73 bp (v2)	
CPEB1	pb 5′ CCCAGAGCAGCACACACTCGGTACTG 3′	358 (v1), 134 (v2)		
PBGD	fw 5′ GCTATGAAGGATGGGCAACT 3′	808 (v1), 756 (v2), 688 (v3), 637 (v4)	149 bp (v1–v4)	NM_000190
PBGD	rev 5′ GTGATGCCTACCAACTGTGG 3′	936 (v1), 886 (v2), 817 (v3), 766 (v4)		
PBGD	pb 5′ TGCCCAGCATGAAGATGGCC 3′	906 (v1), 855 (v2), 786 (v3), 735 (v4)		

“fw”and “rev” mark forward and reverse primers, “pb” probe and “v” transcript variant.

Supplementary Table S4: Primers used for reverse transcription and identification of alternative CPEB1-4 splice isoforms

Gene	*Sequence*	*Position*	*Predicted product length*	*GeneBank accession no.*	*Detected product length*	*Isoform*
CPEB1	fw 5′ GGATTGGTTAACACCTTCCGTG TTTTTGGC 3′	967 (v1),				
		751 (v2)	176 bp (v1)	NM_030594		
CPEB1	rev 5′ AGGCCATCTGGGCTCAGCGGG 3′	1131 (v1),				
		921 (v2)	191 bp (v2)	NM_001079533		
					169 bp	CPEB1Δ5
					182 bp	CPEB1
CPEB2	fw 5′ AACTCCATCACTGACTCCAAAATCT 3′	1860				
CPEB2	rev 5′ CAAGCCATCATCTATTGGAA AGAGGGAAGA 3′	2206	375 bp (v4)	NM_001177382	375 bp	CPEB2a
		2182	352 bp (v2)	NM_182485	351 bp	CPEB2b
		2125	295 bp (v3)	NM_001177381	294 bp	CPEB2 c*
		2116	286 bp (v5)	NM_001177383	286 bp	CPEB2c
		2101	271 bp (v6)	NM_001177384	271 bp	CPEB2d*
		2092	262 bp (v1)	NM_182646	262 bp	CPEB2d
CPEB3	fw 5′ CAAAAAGCCCTTCTCCAGCAAC 3′	876				
CPEB3	rev 5′ TTCAGCTTTGTGAGGCCAGTCTA 3′	14578	604 bp (v1)	XM_006717715	600 bp	CPEB3a
		1433	580 bp (v3)	XM_011539514	576 bp	CPEB3b
		1388	535 bp	*	531 bp	CPEB3c
		1364	511 bp	*	507 bp	CPEB3d
CPEB4	fw 5′ CAGCTCTGCCTTTGCACCTAAAT 3′	1053				
CPEB4	rev 5′ GGCCATCATCCAAGAATCCATC 3′	1309	278 bp (v1)	NM_30627	277bp	CPEB4a
		1286	255 bp	*	254bp	CPEB4b
		1258	227 bp (v2)	NM_001308189	226bp	CPEB4c
		1234	203 bp (v3)	NM_001308191	203bp	CPEB4d

*constructed based on a transcript variant alignment,

The isoforms of CPEB2 were described according to Turimella et al., 2015.

Supplementary Table S5: RT-PCR products (relative fluorescent units) of splice variants of the CPEB 1-4 genes

A

The RT-PCR products of specific splice isoforms of the *CPEB1* gene:

Detected alternative splice isoforms:		Δ5			
**Diagnose**	**ID/T-Nummer**	**169 bp**	**182 bp**	**508 bp**	**599 bp**
AAIII	2377	26683			
AAIII	2526	30894			
AAIII	2725	21376			
AAIII	2744	23806			
AAIII	2771	31813			
AAIII	2897	37449			
AAIII	2899	24278			
AAIII	3423	54168			
AAIII	3545	28195			
AAIII	3546	45194			
AAIII	3548	34216			
AAIII	4045	32681			
GBM	71	38072			
GBM	72	32832			
GBM	132	27337			
GBM	172	21483			
GBM	327	7186			
GBM	328	25658			
GBM	625	27745			
GBM	862	19256			
GBM	1010	25856			
GBM	1311	30762			
GBM	1619	20905			
GBM	1968	26145			
GBM	2010	34031			
GBM	2104	23173			
GBM	2169	18711			
GBM	2304	25799			
GBM	2481	27551			
GBM	2486	26420			
GBM	2494	30091			
GBM	2735	43101			
GBM	2757	20864			
GBM	2854	30034			
GBM	2884	21244			
GBM	2896	19339			
GBM	3007	52052			
GBM	3031	37154			
GBM	3032	26770			
GBM	3066	7859			

GBM	3070	16581	**1909**		
GBM	3513	30871			
GBM	3747	36070			
GBM	2655	20061			
GBM	3527	21366			
GBM	2643	17833			
GBM	3555	18439	**2160**		
**normal brain**	**frontal**	**17581**			
**normal brain**	**occipital**	**14549**			
**normal brain**	**parietal**				**1864**
**normal brain**	**temporal**	**24982**			
OAII	1418	26306			
pGBM	820	38785			
pGBM	176	24154			
sek GBM	677	18769		**3412**	
sek GBM	1214	24461			
sek GBM	1329	39085			
sek GBM	1430	24163			
sek GBM	1944	16999			
sek GBM	2007	43109			
sek GBM	2727	20646			
sek GBM	3475	17974			
GBM cells	A172	38776			
GBM cells	LN229	20089			
GBM cells	LN428	27441			
GBM cells	T98G	23426			
GBM cells	U178	48217			

Splice isoforms present in normal brain tissue are marked by bold font.
Splice isoforms more abundant in glioma tissue and reduced or lacking in normal brain tissues are marked by italics bold font.

B
The RT-PCR products of specific splice isoforms of the *CPEB2* gene:

**Detected alternative splice isoforms:**				**2d**	**2d***	**2c**	**2c***	**2b**	**2a**
**Diagnose**	**ID/T-Nummer**	**170 bp**	**203 bp**	**262 bp**	**271 bp**	**286 bp**	**294 bp**	**351 bp**	**375 bp**
AAIII	2377			***19243***	***3816***	21200		***7022***	8456
AAIII	2526			***15307***		28051	4055		4453
AAIII	2725			***13604***	***2454***	17950	2852	***3193***	4612
AAIII	2744			***7681***		12972			4560
AAIII	2771			***8959***	***1071***	13648	1525	***1776***	2961
AAIII	2897			***9514***		34779	4853	***2798***	16075
AAIII	2899			***5384***		24686	4201		8263
AAIII	3423			***9284***		23739	2808		6835
AAIII	3545			***19103***		31827	3676	***4930***	10797
AAIII	3546			***9080***		34540	6921		7906
AAIII	3548			***12417***		23140	2490	***3353***	7882
AAIII	4045			***3934***		57221	4731		9744

GBM	71			***15391***	***2816***	5999		***1907***	
GBM	72			***23004***	***3795***	11148		***5366***	
GBM	132			***8845***		9613		***1354***	
GBM	172			***16565***		15177			5853
GBM	327			***19305***		5559		**2000**	
GBM	328			***27750***		20949			6870
GBM	625			***28744***	***3612***	10825		***7319***	3753
GBM	862			***17776***	***2447***	6002		***1722***	
GBM	1010			***15453***	***2242***	9012	1193	***3549***	2778
GBM	1311			***39092***	***4376***	12895		***5148***	
GBM	1619			***20667***		14643			
GBM	1968			***22959***		18904		***6162***	
GBM	2010			***21862***	***2844***	16371	2312	***7560***	7230
GBM	2104			***20673***	***2917***	32529	4586		4254
GBM	2169			***33190***	***4799***	10029		***4174***	
GBM	2304			***20938***	***3913***	8495		***2218***	
GBM	2481			***21328***		23116			6976
GBM	2486			***23707***		19784	3520		
GBM	2494			***10869***		12771		***2577***	3845
GBM	2735			***30423***	***5149***	16458		***11587***	8431
GBM	2757			***20331***	***3224***	11469		***5863***	3850
GBM	2854			***16473***	***3507***	31545	6160		4135
GBM	2884			***22520***	***3867***	11169		***3910***	2412
GBM	2896			***18772***	***3692***	21197			5594
GBM	3007			***35081***	***6034***	25479	4176	***7502***	7393
GBM	3031	422	567						
GBM	3032			***23106***	***4075***	21910	3737	***5109***	5755
GBM	3066			***7374***		4665		***966***	
GBM	3070			***11779***		9861			
GBM	3513			***15812***	***2648***	13211	1889	***3364***	3997
GBM	3747			***3935***		35837	4589		9197
GBM	2655			***5707***		16105	3979		
GBM	3527			***19454***	***3971***	16649	3240	***2610***	2390
GBM	2643			***15336***		9016			
GBM	3555			***15783***	***1412***	8804		***4047***	2729
**normal brain**	**frontal**					**19221**	**2402**		**9947**
**normal brain**	**occipital**					**17043**	**2654**		**8193**
**normal brain**	**parietal**					**27201**	**3259**		**15137**
**normal brain**	**temporal**					**9943**			**5267**
OAII	1418			***10382***		27422	3413		9416
pGBM	820			***18678***		17925			
pGBM	176			***13646***		6486			
sek GBM	677			***22717***	***4536***	16365		***6843***	5106

sek GBM	1214			***14151***		22018			
sek GBM	1329			***25454***	***4341***	13992	2448	***3946***	3066
sek GBM	1430			***8732***		13920			2573
sek GBM	1944			***2784***		1408			
sek GBM	2007			***25122***		13291		***5809***	4136
sek GBM	2727			***13130***		13388	2144		
sek GBM	3475			***9065***		25518	4897		6035
GBM cells	A172			***31735***	***3767***	4119		***3787***	
GBM cells	LN229			***22869***	***5466***	1544		***2998***	
GBM cells	LN428			***32980***	***3384***	8621			
GBM cells	T98G			***43905***				***3575***	
GBM cells	U178			***48671***					

Splice isoforms present in normal brain tissue are marked by bold font.
Splice isoforms more abundant in glioma tissue and reduced or lacking in normal brain tissues are marked by italics bold font.

C

The RT-PCR products of specific splice isoforms of the *CPEB3* gene:

Detected alternative splice isoforms:															3d	3c		3b	3a
**Diagnose**	**ID/T-Nummer**	**100 bp**	**104 bp**	**122 bp**	**135 bp**	**143 bp**	**182 bp**	**203 bp**	**254 bp**	**306 bp**	**339 bp**	**416 bp**	**437 bp**	**452 bp**	**507 bp**	**531 bp**	**547 bp**	**576 bp**	**600 bp**
AAIII	2377														14978			7471	
AAIII	2526														16481			5174	
AAIII	2725														8565			5827	
AAIII	2744								1336						8779			3911	
AAIII	2771									2691					11567				
AAIII	2897														10303	3297		7329	6800
AAIII	2899														9728			5401	1662
AAIII	3423														8332			7130	3319
AAIII	3545														10263	1937		6631	5312
AAIII	3546														10090			7658	
AAIII	3548			**2244**		950									14927			5418	1641
AAIII	4045					2809		967	1267						6986				
GBM	71			975											2644				
GBM	72				890										12224			3767	
GBM	132														2960				
GBM	172											2041	1151					5473	
GBM	327													***4332***					
GBM	328														11773			3715	
GBM	625			***3411***				1331							9924			4181	
GBM	862			2902											4307				
GBM	1010	2482													3189			3926	
GBM	1311			1697											5664			4930	
GBM	1619														8481			2164	
GBM	1968							1309							12571			3929	
GBM	2010			1832											11890		4069	5785	
GBM	2104			1684											9475			5370	
GBM	2169	2010		***2775***				2038	1369						7456				

GBM	2304			***3064***											6016			5035	
GBM	2481														4655			1948	
GBM	2486														13216			5885	
GBM	2494														7194			4258	
GBM	2735			***3702***											19438			6404	
GBM	2757														7269			3233	
GBM	2854			***2848***											11950			4230	
GBM	2884			906										***2270***	3166				
GBM	2896														8291			3573	
GBM	3007			***3215***				1769							12083	2690		8418	4942
GBM	3031							1302						1394	13680	2416		5531	
GBM	3032				1488			1435							11285			5442	
GBM	3066			937											5000				
GBM	3070			***2843***							1775				2916				
GBM	3513				1274			958							7020			5493	
GBM	3747								1060						13025			5380	
GBM	2655														6427			5669	
GBM	3527														14338			8036	
GBM	2643				1052										2988				
GBM	3555						1790								7409			4267	
**normal brain**	**frontal**														**2786**			**3347**	**6109**
**normal brain**	**occipital**														**2747**	**1313**		**3014**	**4169**
**normal brain**	**parietal**														**2224**			**4410**	**8886**
**normal brain**	**temporal**														**2423**				**4662**
OAII	1418														10694			3442	
pGBM	820				781										14046			9726	
pGBM	176														1821			994	
sek GBM	677			1794											16129			5345	
sek GBM	1214														10861			4527	
sek GBM	1329														5077			3081	
sek GBM	1430														2564			1991	
sek GBM	1944																		
sek GBM	2007			1156											12615			5049	
sek GBM	2727														3751			2626	
sek GBM	3475														12062			4703	
GBM cells	A172	***4055***		4301											8985			4284	
GBM cells	LN229														2498			2219	
GBM cells	LN428														8673			5298	
GBM cells	T98G	1685		1524															
GBM cells	U178	***9331***	1442	***10162***				1469							9216			3498	

Splice isoforms present in normal brain tissue are marked by bold font
Splice isoforms more abundant in glioma tissue and reduced or lacking in normal brain tissues are marked by italics bold font
D
The RT-PCR products of specific splice isoforms of the *CPEB4* gene:

Detected alternative splice isoforms:					4d	4c	4b	4a			
**Diagnose**	**ID/T-Nummer**	**115 bp**	**142 bp**	**182 bp**	**203 bp**	**226 bp**	**254 bp**	**277 bp**	**306 bp**	**507 bp**	**547 bp**
AAIII	2377				21149		21655				
AAIII	2526				19619		19174			1675	
AAIII	2725				26046		20165				
AAIII	2744				21718		25345				
AAIII	2771				16637		12207		7133		
AAIII	2897				26150	5221	22566	4984			
AAIII	2899				19046	1503	15869	1628			
AAIII	3423				9538		8597	1103			
AAIII	3545				19463		25298				
AAIII	3546				22665		20903				
AAIII	3548				21383		22958	2407		2125	
AAIII	4045				26882		32885	2864			
GBM	71				7320		4185				
GBM	72				15172		15587				
GBM	132				6518		5536				
GBM	172				17460	4028	9620	6182			
GBM	327				12086		8460				
GBM	328				25485		21644				
GBM	625			2634	30452		11702				
GBM	862		3500		15071		5445				
GBM	1010				22305		14188				
GBM	1311				22087		17696				
GBM	1619				14804		15511				
GBM	1968				33990		34922				
GBM	2010				22114		21939				***10977***
GBM	2104				20095		12555	2397			
GBM	2169				49763		37272				
GBM	2304				16788		13189				
GBM	2481				14181		14563				
GBM	2486			1526	25177		22191			1216	
GBM	2494				13383		10394	2237			
GBM	2735				22615		30332			2482	
GBM	2757				13014		16528				
GBM	2854	1810			27502		17757				
GBM	2884				9538		11158				
GBM	2896				21392		15304	2357			
GBM	3007				40036		32132				
GBM	3031				35312		23592				
GBM	3032				37298		26434				

GBM	3066				8825		7801				
GBM	3070				9167		6748				
GBM	3513				21270	2580	19710	3011			
GBM	3747				21631		25574				
GBM	2655				11793		9233	2176			
GBM	3527				22106		22278				
GBM	2643				18032		12070				
GBM	3555			4261	11334		9271	5636			
**normal brain**	**frontal**				**9860**	**6940**	**6026**	**6231**			
**normal brain**	**occipital**				**8343**	**8381**	**5602**	**7068**			
**normal brain**	**parietal**				**20994**	**14305**	**11481**	**10482**			
**normal brain**	**temporal**				**9343**	**7008**	**5371**	**5927**			
OAII	1418				19617		20652				
pGBM	820				20257		13442			1764	
pGBM	176				9898		5532				
sek GBM	677				17046		17065			1621	
sek GBM	1214				16456	2722	14162	2717			
sek GBM	1329				6742		4778				
sek GBM	1430				15947	2555	9824	2453			
sek GBM	1944				8971		3747				
sek GBM	2007				18669		15918				
sek GBM	2727				17089		10782				
sek GBM	3475				11656		16539				
GBM cells	A172				20800		12084				
GBM cells	LN229				14344		10688				
GBM cells	LN428				15692		12416				
GBM cells	T98G				27548		7879				
GBM cells	U178				38061		17132				

Splice isoforms present in normal brain tissue are marked by bold font.
Splice isoforms more abundant in glioma tissue and reduced or lacking in normal brain tissues are marked by italics bold font.
Fragment analysis of CPEB alternative splice isoforms in AAIII, pGBM, sGBMs, glioblastoma/medulloblastoma cell lines and normal brain tissue. The values above the table correspond to the RT-PCR product (size in basepairs) generated by fragment analysis.
